# An Essay on the Physiology of the Iris

**Published:** 1834-04-01

**Authors:** 


*An Essay on the Physiology of the Iris.* By JOHN WALKER, Assistant Surgeon to the Manchester Eye Institution, &c.—Manchester, 1833.—8vo. pp. 16.

THIS pamphlet does credit to the talents and physiological acumen of its ingenious author. It will be sufficient to quote the last few sentences, as they contain a summary of the whole.

"Upon a review of the whole of the facts and arguments adduced, it appears to result, first, that the motions of the iris depend, not upon impressions made on the retina, but upon its own sensibility to light, as also upon its association with the muscles of the eyelids, through the medium of the lenticular ganglion: second, that these motions are for the purpose of adapting the eye to the perception of objects at different distances: third, that the iris acts also in some measure as a defence to the more internal parts of the eye, as the palpebræ do to the external, forming, in fact, an internal eyelid: fourth, that the external parts of the eye generally, as well as the iris, are sensible to the stimulus of light;* and lastly, that the retina is insensible to the ordinary impressions of light, as a stimulant; but that its functions may be impaired or destroyed by the more powerful concentration of light, as from the sun's rays, the *coup de soleil*, or from lightning, in the same manner as the brain and other parts of the nervous system are injured or paralyzed from the like causes." (P. 16.)

